# Seminatural environments for rodent behavioral testing: a representative design improving animal welfare and enhancing replicability

**DOI:** 10.3389/fnbeh.2023.1192213

**Published:** 2023-06-22

**Authors:** Enrique Hernández-Arteaga, Anders Ågmo

**Affiliations:** ^1^Human Development Faculty, Autonomous University of Tlaxcala, Tlaxcala, Mexico; ^2^Department of Psychology, University of Tromsø, Tromsø, Norway

**Keywords:** replicability, external validity, animal welfare, seminatural environments, generalizability

## Abstract

The low replicability of scientific studies has become an important issue. One possible cause is low representativeness of the experimental design employed. Already in the 1950’s, Egon Brunswick pointed out that experimental setups ideally should be based on a random sample of stimuli from the subjects’ natural environment or at least include basic features of that environment. Only experimental designs satisfying this criterion, representative designs in Brunswikian terminology, can produce results generalizable beyond the procedure used and to situations outside the laboratory. Such external validity is crucial in preclinical drug studies, for example, and should be important for replicability in general. Popular experimental setups in rodent research on non-human animals, like the tail suspension test or the Geller-Seifter procedure, do not correspond to contexts likely to be encountered in the animals’ habitat. Consequently, results obtained in this kind of procedures can be generalized neither to other procedures nor to contexts outside the laboratory. Furthermore, many traditional procedures are incompatible with current notions of animal welfare. An approximation to the natural social and physical context can be provided in the laboratory, in the form of a seminatural environment. In addition to satisfy the basic demands for a representative design, such environments offer a far higher level of animal welfare than the typical small cages. This perspective article will briefly discuss the basic principles of the generalizability of experimental results, the virtues of representative designs and the coincidence of enhanced scientific quality and animal welfare provided by this kind of design.

## Introduction

The use of non-human animals for modeling human behavior, disease, and other conditions is based on the premise that human situations can be recreated in these animals. However, most animal models focus on a particular aspect of the situation to be modeled, without holistically considering the processes that occur neither in humans nor in the species used for modeling the human condition. However, the behavioral patterns displayed vary depending on the characteristics of the environment in a species-specific way (Fierro Toscano and Andrade, 2016). Ignoring the subtle interaction between environment and organism when studying behavior may have costly consequences.

The aims of the present article are to briefly introduce what has been labeled the replicability and generalizability crisis as well as the low predictive value of many animal studies. Then we will propose that a different kind of design, representative design, could enhance replicability and generalizability of animal models of human conditions, thereby improving the predictive value. It will also be mentioned that considerations of animal welfare were not given any fundamental importance in many of the established animal tests. We will argue that a representative design, in the form of a seminatural environment, much improves animal welfare.

Before entering into the specific subjects of the present contribution we need to define some basic concepts, including the distinction between animal models and animal tests. Some have defined “tests as behaviors that can be evaluated, whereas an animal model is an animal that has been manipulated as to score higher in these tests” (Söderlund and Lindskog, 2018, p. 669). Others use “model” as synonym to “test,” i.e., a specific procedure aimed to predict the effects of a manipulation, for example the administration of a drug, in humans suffering from disease or dysfunction (Cryan et al., 2005; Planchez et al., 2019). In the article, we use behavioral test when referring to the exposure of an organism to a specific situation in order to assess a behavioral variable of interest and behavioral model when a behavior pattern of an organism is considered as representative of the behavior of another, generally more complex, organism.

### The reliability of scientific studies

Replicability, repeatability, or reproducibility refer to the likelihood of obtaining similar results with a new dataset in a procedure identical or similar to the procedure used in the original study (Kenett and Shmueli, 2015; Patil et al., 2019). The low replicability of scientific studies has been of concern for many years. It has been suggested that more than half of the claims made in scientific publications are false (Ioannidis, 2005). Low replicability has been reported for the neurosciences (Button et al., 2013) as well as the medical (Prinz et al., 2011) and social sciences (Camerer et al., 2018), including psychology. In fact, several failed intents to replicate landmark studies in psychology (Open Science Collaboration, 2015; Wagenmakers et al., 2016) originated a phenomenon labeled “the replicability crisis.”

The remedies for low reproducibility is thought to be enhanced scientific rigor, meaning that, for example, statistical methods should be strengthened, the analysis plan should be prepublished, collaboration across labs should be stimulated, data should be made openly available, and detailed experimental procedures should be reported (Munafo et al., 2017; Stevens, 2017; Ganley et al., 2022; Lu and Daugherty, 2022).

### The predictive value of studies in non-human animals

If we are testing drugs in non-human animals with the purpose to predict clinical effects in humans, we are not only facing a replicability problem but also questions concerning the validity of the test. This becomes especially evident if the test is intended to represent a human psychopathology such as depression, anxiety or schizophrenia, or one of the sexual dysfunctions. Since these conditions have no equivalent in non-human animals, suppositions must be made concerning the correspondence between the behavior expressed in the animal test and the alterations observed in human psychopathology. These suppositions are often questionable. Indeed, whether popular rodent tests of anxiety, like the elevated plus maze, the open field or the dark/light transition test really represent the human anxiety condition (Ennaceur, 2014; Ennaceur and Chazot, 2016) or if they have any predictive validity or not (e.g., Rosso et al., 2022) are subjects of endless debates. The same is the case for other animal tests designed to be representative of human mental disease (Commons et al., 2017; Białoń and Wąsik, 2022). Thus, as soon as animal behavior is used as a model for human psychopathology, besides the problems of replicability, we have the quandary of the validity of the animal model itself. To these difficulties we have to add the uncertainty of generalizations from one species to another.

In the last sentence of the preceding paragraph, generalization means the extent to which the behavioral effects of an experimental manipulation, such as drug treatment, obtained in one species also would occur in other species. This is different from the use of the term generalization in statistics. There, it refers to whether the effects found in a random sample are applicable to the population from which the sample was drawn. There are many vicissitudes even in this kind of generalization, and a generalizability crisis in inferential statistics is presently of considerable concern (Yarkoni, 2022). A third kind of generalization refers to the applicability of results obtained under strictly controlled laboratory conditions to situations outside of the laboratory.

The generalization of effects observed in one species to another species combined with generalizations from the experimental conditions used in the preclinical studies to effects in the clinic is apparently not particularly successful. About 90% of all clinical drug trials fail, even though they are based on the best available animal data (Sun et al., 2022). The success rate is particularly low for CNS active drugs (6.3% vs. 13.3% for non-CNS drugs; Gribkoff and Kaczmarek, 2017). The dismal predictive validity of the preclinical studies made most established pharmaceutical companies in Europe, Japan, and the US to shut down their CNS research facilities many years ago (Abbott, 2011).

The problems of replicability within a species and the poor generalizability of effects from one species to another combined with the uncertainty concerning the validity of the animal model may seem unsurmountable. Over the years, many solutions have been offered (e.g., Meyerson and Lindström, 1973; Olivier et al., 1990; Peters et al., 2015; Kafkafi et al., 2018; Storey et al., 2021), but their success has been limited or non-existent since none of these problems has been eliminated. However, the recent proposal (Voelkl et al., 2020, 2021) that systematic incorporation of confounding factors, leading to “controlled heterogenization” would improve external validity and reproducibility is interesting. The complicated statistical procedures and large samples required for this approach may reduce its feasibility, though. Nevertheless, data suggest that heterogenization indeed improves replicability and generalizability, at least in animal models of ischemic stroke (Usui et al., 2021).

It is possible that an entirely different kind of experimental design, involving holistic considerations about the processes that occur both in humans and in the species used for modeling the human condition, might improve generalizability within a species as well as applicability to context outside of the laboratory. Indirectly, it might even improve interspecies generalizations, and perhaps enhance the validity of the animal models.

### Animal welfare

Besides the many problems outlined above, studies in animals have been criticized because of concerns for animal welfare (e.g., Brown and Winnicker, 2015; d’Isa and Gerlai, 2023). These concerns are not necessarily related to worries about scientific reliability, but they acquire additional weight when it is pointed out that a substantial part of the scientific effort is wasted because of lack of reliability and clinical relevance. It has been claimed that about 28 billion US$ are spent on irreproducible research every year in the United States alone (Freedman et al., 2015). Provided that some studies require that animals are subjected to varying levels of discomfort, it can be argued that the discomfort inflicted on them is pointless since the data obtained may be both unreliable and without clinical relevance, despite claims to the contrary (see Stanford, 2020, for an excellent discussion). The social standing of science would be much improved if we could develop experimental setups assuring some degree of welfare for the subjects and a high degree of replicability and generalizability, including to the clinic.

### The problems with standard behavioral tests

In standard behavioral tests for laboratory rodents, animals are housed in home-cages and their behavior is evaluated in specific test sessions, performed outside the home-cage, which last generally between a few minutes and 1 h. There has been a long tradition to design such experimental procedures so that the animals’ behavioral repertoire becomes as limited as possible. For example, when studying learning, be it in a T-maze, in a Skinner box, or on a radial maze, the researcher tries to eliminate all stimuli that are considered irrelevant, thereby avoiding distractions that might perturb the animals’ performance. Odors are normally eliminated from the setup, unnecessary visual stimuli likewise, and sounds can either be reduced as much as possible or masked by a white noise. The response options are also limited to what is considered of interest, like running in the aseptic runway of the maze and turning either to the left or the right, or pressing the manipulandum, or walking back and forth on the arms of the radial maze. In the case we study sexual behavior, a heterosexual couple is enclosed in a barren arena where they can choose between sleeping, fighting, or copulating. In the Porsolt test, the options are to try the impossible escape or give up and drown. In sum, the setup is arranged in such way that there are no distracting stimuli and few response options. This experimental ideal was brilliantly exposed by American psychologist Kenneth Spence (1907-1967) in his classic 1956 book (Spence, 1956).

The approach described in the preceding paragraph is excellent for hypothesis testing, and is often labeled systematic design (Brunswik, 1947). Since the experimental subjects’ behavioral repertoire has been limited to the behaviors of interest and since irrelevant stimuli have been eliminated, at least as far as possible, the systematic design is a powerful tool to test specific hypothesis.

### The notion of representative design

A key notion in experimental design is that the experimental subjects should be a random sample of the population. If the experimental groups were not composed according to this notion, all the statistical tests now being an integral part of any scientific endeavor would be meaningless, because they are all based on the assumption of a random sample. The results obtained in the sample can be generalized to the population from which the sample was drawn only if the sample was random. It is common to talk of a representative sample, when special care has been taken in the sampling procedure.

In addition to the requirement of a random sample of subjects, it has been suggested that the experimental design should include random samples of potentially relevant variables or of procedures appropriate for evaluating the research question (Petrinovich, 1989; Dhami et al., 2004; Araujo et al., 2007; Scholz, 2017). Such a design would be labeled “representative design.” According to the Brunswikian notions, it would not be sufficient to include additional subject variables such as sex, age, degree of deprivation, etc. Variations of context (procedure) are an indispensable part of a representative design.

The term was originally proposed by the psychologist Egon Brunswik (1903 – 1955). Although forgotten by many young psychologists, Brunswik was quite influential in the 1950’s and for several years thereafter. He was of Hungarian origin, educated in Vienna, where he got his Ph.D. in psychology in 1927. In 1937, he moved to Berkeley where he remained until his death in 1955. During his time in Vienna, Brunswik occasionally participated in the Vienna circle, a group of neopositivist philosophers animated by German philosopher and physicist Moritz Schlick (1882 – 1936) and including, among others, Austrian mathematician and logician Kurt Friedrich Gödel (1906 – 1978), Austrian philosopher and sociologist Otto Neurath (1882 – 1954) and German philosopher Rudolf Carnap (1891 – 1970). The emphasis on the logical foundations of knowledge and theory construction typical of the Vienna circle are basic to Brunswik’s ideas (Leary, 1987). Brunswik believed that humans and animals live in environments that are chaotic and constantly changing. Certain stimuli in the environment are reliable predictors of important events and are considered ecologically valid in brunswikian terms. Most stimuli have no predictive value and can be safely ignored. Brunswik’s famous double lens model (Brunswik, 1955) provides an illustration of how ecologically valid stimuli function. In order to determine the ecological validity of a stimulus, the stimulus needs to be evaluated in a representative design in the sense described in the preceding paragraph.

Even though the concept of representative design originated in studies of perception, it can be applied to any field of behavioral inquiry. If we are interested in finding out if a drug has antidepressant properties in preclinical tests, for example, we have many test procedures to choose from. A recent review of the most used current animal tests of major depression listed more than 20 (Planchez et al., 2019). Actually, the total number of tests supposed to represent depression is far larger than that. Thus, it can be maintained that there is a population of tests for studying major depression. According to the notion of representative design, we should draw a random sample from that population, and then use all the sampled tests in our experiment. Such a random sample of test procedures would assure that our results can be generalized to the entire population of test procedures usable for testing antidepressant drugs.

In practice, a representative design as described here is cumbersome and extremely costly. It has been suggested that an acceptable approximation could be to introduce crucial elements of the subject’s natural habitat in the experimental setup (Petrinovich, 1980).

In humans, rather than introducing elements from the habitat into the laboratory, experiments can be performed outside the laboratory. In fact, there are many recent examples of research performed in people’s natural environment (Sliwinski et al., 2018; Richmond and Burnett, 2022). We will not further discuss the application of representative design to studies in humans, but we find it important to mention that it is quite feasible. Instead, we will focus on designs suitable for experiments in rodents.

There are fields of inquiry that would not benefit from the use of representative designs. Many physiological processes, like water reabsorption in the loop of Henle, or the release of thyroid stimulating hormone in response to cold, can be adequately studied without any representative design. In fact, such designs are relevant particularly in behavioral studies. Nevertheless, even in behavioral experiments, they may not always be needed. The molecular mechanisms involved in estradiol’s facilitation of lordosis may be perfectly understood by using extremely simple designs, and the results are generalizable to all contexts in which lordosis is displayed. They are also perfectly replicable (Pfaff, 2017). Whether they can be generalized from rats to women is an entirely different question, particularly since lordosis is not a basic part of sexual behavior in women.

It is mainly when complex behavioral phenomena are the subject of study that representative designs become crucial. This is also the case when hypotheses about the adaptive value or biological functions of behavior are to be made.

### Seminatural environments as representative design

If the aim of an experiment is to determine the effects of a drug or of a manipulation of the brain on behavior, then procedures like the Porsolt test are entirely unsuitable. In experiments with this wide purpose, we need to employ a design allowing the experimental subjects to express as much as possible of their behavioral repertoire. Then we could even see whether the drug or manipulation has any unexpected or novel effects, i.e., we would make discovery research (Foletti and Fais, 2019). Preferably, this should be done in an environment offering a rich variation of stimuli acting on several sensory modalities. Ideally, a complete ethogram should be established, and the observation time should be long enough to record several occurrences of relevant behavior patterns. Modern computational techniques have reached a stage in which complex behavior patterns can be automatically identified and described in excruciating detail, even when several animals are observed simultaneously (Egnor and Branson, 2016; Kennedy, 2022; Lauer et al., 2022). This amazing progress makes the kind of studies mentioned above feasible without excessive investment of labor.

**FIGURE 1:**
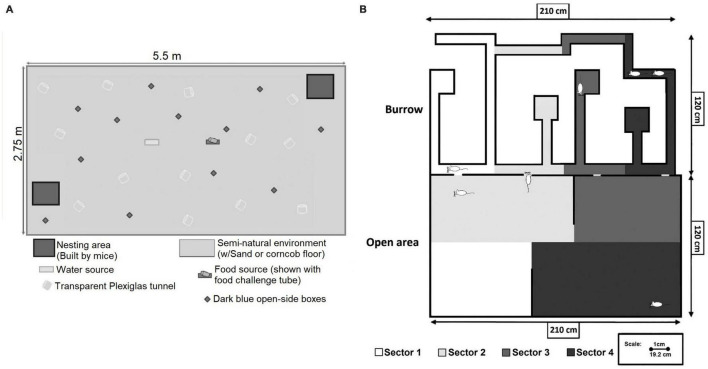
**(A)** The seminatural environment used in studies in mice in the Pfaff laboratory at the Rockefeller University. It consisted of a large space with food and water sources, as well as materials for nest building. For a detailed description, see Ragnauth et al. (2005) from which the figure is reproduced with permission from Wiley. **(B)** The seminatural environment used in the Ågmo laboratory at the University of Tromsø. It consisted of complex burrow system and a large open area. For analysis of localization of behavior, the environment was divided into four sectors. Reprinted from Le Moëne and Ågmo (2018) with permission from Elsevier. Further details can be found in that paper as well as in Chu and Ågmo (2014).

A way to combine the requirements of a representative design and discovery research is to create a complex test environment allowing the subjects to express as much as possible of their natural behavioral repertoire. This becomes possible when the basic features of the natural habitat are preserved in the experimental procedure. There are several examples of experimental setups in animal research that satisfy these demands (e.g., McClintock, 1981; Blanchard et al., 1995, 2001; Ragnauth et al., 2005; Weissbrod et al., 2013). The Ragnauth et al. (2005) environment, employed at the Rocckefeller University, is illustrated in Figure 1A. In rats, the essential features are the presence of several conspecifics, the availability of something similar to a burrow, and a reasonably large physical space. Studies of wild rats have systematically shown that several individuals share a burrow, that they are sociable and that sexual interactions involve several individuals (Barnett, 1958a; Calhoun, 1962; Robitaille and Bovet, 1976; Schweinfurth, 2020). In agreement with this, at the University of Tromsø we built a two-dimensional copy of a rat burrow (Figure 1B), based on data from Calhoun (1962) and on the seminatural environment described by McClintock and Adler (1978). The burrow was connected to a large open field. Lighting was so arranged that the burrow was kept in constant darkness for the rats, but illuminated with infrared light (850 nm) for the video cameras. The open field had a day beginning and ending with a 30 min period of increasing and decreasing light intensity, respectively, simulating sunrise and dusk. During the night, the light intensity was about 10 lx at floor level, not much different from the light provided by a full moon. Experiments lasted 8 days, and groups of 4 female and 3 male rats were always used. The sex ratio is close to what is found among adult rats in nature. Since the environment include the basic features of the natural habitat, we consider it appropriate to call it seminatural. Detailed descriptions of this environment can be found elsewhere (Chu and Ågmo, 2014, 2015b).

It is important to note that careful studies have revealed that laboratory rats share most behavioral characteristics with wild rats (Boice, 1977, 1981; Flannelly and Lore, 1977; Price, 1980), the main exception being that wild rats are far more neophobic than laboratory rats (Barnett, 1958b). However, there are also data showing that wild rats captured in an urban environment are not more neophobic than laboratory rats (Koizumi et al., 2021). Thus, we maintain that the seminatural environment is as valid for laboratory rats as it would be for wild rats, and that observations in this environment can be generalized to the natural habitat.

Descriptions of sociosexual interactions in this environment have revealed a considerable number of features that had not been detected in standard tests of sexual behavior, performed in heterosexual couples in a small observation arena. Among these are the sudden transition from non-receptivity to full receptivity at the beginning of behavioral estrus (Chu and Ågmo, 2015a; Le Moëne et al., 2020a). In the standard observation environment, in which the female has no escape from a sexually active male, the transition is gradual. Another feature not evident in the standard environment is that males and females equally control the sexual interactions (Bergheim et al., 2015). Indeed, seminatural environments provide the female with ample opportunities to control sociosexual interactions, at difference to most standard environments in which the male appears to dominate (Chu and Ågmo, 2014, 2023). The fact of providing the females with these opportunities, reproducing the situation occurring in nature, makes seminatural environments a more realistic model of biredirectional socio-sexual interactions between males and females. In addition, it also makes seminatural environments research tools more suitable for the welfare of the female subjects.

In the studies mentioned in the preceding paragraph, as well as in many others, the purpose was to understand the dynamics of rat sexual behavior, without the slightest intention to generalize the results to other species. What we pretended, though, was to be able to generalize our findings to rat behavior outside the laboratory. Valid generalizations to the natural habitat make it possible to present fruitful analyses of the adaptive value of behavior patterns, rather than the sterile speculations based on data from standard procedures lacking external validity.

Seminatural environments are useful not only for detailed descriptions of animal behavior, but they can also be used in experiments. Early examples were the introduction of a predator (a cat) in the open area of the visible burrow system in studies of defensive behavior (Blanchard and Blanchard, 1989; Blanchard et al., 1991). More recent examples are studies on the role of the estrogen receptors α and β in several hypothalamic nuclei in female sociosexual interactions (Snoeren et al., 2015). In our laboratory, we have also introduced different kinds of events in the environment. Among what we believe to be emotionally positive events are the odor of lavender, the sudden availability of chocolate pellets or the sound of a sonata by Mozart. Emotionally negative events can also be used, for example a strong white noise or fox odor (Le Moëne and Ågmo, 2018; Le Moëne et al., 2020b). The behavioral consequences of these events can then be described in untreated rats, in rats where hormone receptors have been manipulated (Le Moëne et al., 2019), in rats treated with anxiogenic or anxiolytic drugs (Le Moëne and Ågmo, 2019), or whatever treatment found of interest. The use of the seminatural environment, i.e., a representative design or a design with external validity, should make it legitimate to generalize the findings to rat behavior in all kinds of situations inside and outside the laboratory. However, while intraspecies generalizations of the results can be made, it would be very risky to maintain that we can generalize to other species. Furthermore, in the studies mentioned above, there was no intention to model human pathologies, and no speculations as to clinical relevance of the results were made. Nevertheless, it has been suggested that procedures based on spontaneous behaviors being part of the natural repertoire are needed for developing valid models of human disorders (Puscian and Knapska, 2022).

Variable environments, for example seminatural environment, are more representative of real biological systems and therefore have greater predictive validity and replicability. However, one of the main challenges that a researcher faces when using variable environments in animal models is precisely the need for better standardization of research protocols and understanding the inherent variability in biological systems, which could reduce the sensitivity of the experimental assessment (Voelkl et al., 2020).

### Employing seminatural environments to model human behavioral and psychiatric disorders

So far, no attempt has been made to use a seminatural environment for describing the behavior of any animal model of human disease. However, in principle this could be extremely helpful. For example, studies of rats prenatally treated with valproic acid, a model of autism (Nicolini and Fahnestock, 2018), in seminatural environments could provide a much richer behavioral characterization than any of the procedures currently used. Such a characterization could be important for a better understanding of the behavioral alterations in autism and provide an opportunity for evaluating treatments. Any of the many transgenic rat strains, supposedly modeling pathologies such as schizophrenia (Uzuneser et al., 2019), Parkinson’s disease (Paldino et al., 2022) or depression (Matthes et al., 2019) could also be studied, just to mention a few examples. There is no doubt that such studies could shed new light on many of the behavioral alterations hitherto poorly understood. This, in turn, may open doors to the neurobiological bases of these alterations.

### Animal welfare in seminatural environments

Quantifications of animal welfare is a tricky issue (Le Moëne and Ågmo, 2017). However, there is consensus concerning the basic importance for animal welfare of having the opportunity to express a substantial proportion of the natural behavioral repertoire (Miller et al., 2020). In fact, compared to standard laboratory tests, seminatural environments offer a high degree of welfare to the animals (Makowska and Weary, 2016). It appears that such environments satisfy most of the recently proposed criteria for animal-friendly tests (d’Isa and Gerlai, 2023). The subjects are allowed to interact with conspecifics while having the possibility to avoid or escape from social contact. Moreover, the subjects are provided with a relatively large and complex space to move in, which gives them the possibility to express a substantial part of their behavioral repertoire. The environmental disturbances introduced in some experiments could be considered part of rats’ natural habitat. While walking around in the garbage dump, rats will be exposed to odors of all kinds, including urine and feces from the cats and dogs in the neighborhood, they can find highly palatable as well as uneatable food, and suddenly be victims of loud noises. All these events, and many more, may occur in rapid succession during any nocturnal walk outside the burrow. They might be aversive, but it is known that rats’ emotional responses to aversive events are attenuated when conspecifics are present (Kiyokawa and Hennessy, 2018; Denomme and Mason, 2022), as is the case in the seminatural environment. Moreover, all aversive stimuli mentioned here are of short duration, and it is known that rats resume their normal activities within less than 5 min after the end of an aversive event, like strong white noise (Le Moëne et al., 2020b). The fact that all events occur in a well-known, safe environment probably contributes to this. Thus, we propose that seminatural environments, in addition to providing a higher number of stimuli positively modulating the affective state of rats, also provide an enrichment buffer which enhances the rats’ resilience to stress and to possible aversive stimuli.

## Conclusion

Seminatural environments not only satisfy requirements for a representative design, thereby assuring external validity and improved replicability, but also enhance animal welfare. The drawback of this kind of environment is the low throughput. Drug screening, for example, would be entirely impracticable in such environments. On the other hand, seminatural environments can be helpful for testing animal models of human psychopathologies and they have recently been proposed as a paradigm that could revolutionize translational psychiatry (Shemesh and Chen, 2023). We have summarized the advantages and disadvantages of seminatural environments in Table 1.

**TABLE 1:** Advantages and disadvantages of seminatural environments.

Advantages	Disadvantages
The data can be generalized to rats’ behavior outside the laboratory, including the natural habitat.	There are no established standards for the design of seminatural environments. Heterogenous conditions might reduce the sensitivity of the experimental assessment.
It is possible to describe, in excruciating detail, the interactions among several animals observed simultaneously.	At present it is unclear whether these environments are helpful for modeling human psychopathologies.
Allow researchers to observe a higher number of fine behavioral features than possible in standard tests.	Demanding in lab space and time investment required for performing experiments as well as for data collection and analysis.
In addition to observational studies, they can be used in naturalistic experimental conditions, for example for evaluating emotion-inducing events occurring in the environment.	Not suitable for high throughput studies.
They offer a higher degree of welfare to animals because they satisfy most of the criteria for animal-friendly tests.	It could be necessary to complement the results obtained with data from additional procedures when modeling human psychopathologies.
They may reduce the number of animals required for appropriate power because of the huge number of data points obtained from each animal.	

The use of seminatural environments has remained at a rather low, stable, level for several decades. However, the enormous progress in automated analyses of videorecorded behaviors, even in group living animals, have made studies in seminatural environments easier to implement and consequently more attractive. Indeed, seminatural environments are a promising tool for both neuroscientific and psychiatric translational research.

## Author contributions

Both authors listed have made a substantial, direct, and intellectual contribution to the work, and approved it for publication.
